# Highlights from Heart Rhythm 2018: Managing Heart Failure—It’s Electric!

**DOI:** 10.19102/icrm.2018.090911

**Published:** 2018-09-15

**Authors:** Imran Niazi

**Affiliations:** ^1^Aurora Cardiovascular Services, Aurora Sinai/Aurora St. Luke’s Medical Center, West Allis, WI, USA; ^2^University of Wisconsin School of Medicine and Public Health, Madison, WI, USA

**Keywords:** Cardiac contractility modulation, CRT, heart failure

The late-breaking trials presented this year at the 2018 Heart Rhythm Scientific Sessions in Boston, MA included two intriguing studies involving the electrical therapy of heart failure.

## FIX-HF-5C

The Evaluate Safety and Efficacy of the OPTIMIZER^®^ System in Subjects with Moderate-to-severe Heart Failure (FIX-HF-5C) study was a randomized, controlled prospective trial of cardiac contractility modulation (CCM) for the treatment of persistent class III/IV heart failure in patients with a narrow QRS and a left ventricular (LV) ejection fraction (LVEF) of 25% to 45%.^1^ CCM is a novel electrical therapy that involves the delivery of a train of electrical impulses during the absolute refractory period of the cardiac cycle. These high-amplitude, biphasic impulses seem to alter intracellular calcium handling, resulting in an increase in myocardial contractility without an associated increase in oxygen consumption. In such a case, the LV pressure–volume loop is shifted to the left. In the long-term, CCM appears to normalize the altered expression of proteins regulating calcium handling and excitation–contraction coupling from the fetal pattern seen in heart failure to the adult pattern seen in normal hearts. These proteins include SERCA2a, phospholamban, the ryanodine receptor, and myosin heavy chain.

**Figure 1:**
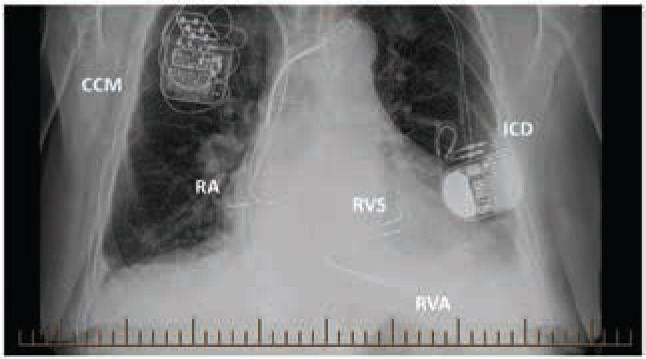
Anteroposterior chest X-ray of a patient with an implanted CCM device and a dual-chamber ICD. CCM: cardiac contractility modulator generator; ICD: implantable defibrillator generator; RVS: CCM ventricular leads placed in the right ventricular septum; RVA: RV apical ICD lead; RA: right atrial ICD and CCM leads.

Electrical stimulation is delivered by the Optimizer^®^ system, (Impulse Dynamics, Mt. Laurel, NJ, USA), which consists of an implantable cardioverter-defibrillator (ICD)-sized, rechargeable pulse generator connected to atrial and ventricular leads **(Figure 1)**. The ventricular leads screw into the right-sided interventricular septum, but the treatment effect extends to the left ventricle.

This system has undergone a series of human trials, with the most recent being FIX-HF-5,^2^ which was completed in 2009. FIX-HF-5 was a randomized open-label study of CCM plus optimal medical therapy versus optimal medical therapy alone in 428 patients with New York Heart Association (NYHA) functional class III/IV heart failure, an LVEF of < 35%, and a narrow QRS. The primary efficacy endpoint was unusual, in that it was mean change in anaerobic threshold during treadmill exercise between the two groups. This was not met. However, there was a statistically significant improvement in peak oxygen consumption (VO_2_), NYHA functional class, and quality of life. A subgroup analysis^3^ of the data from patients with NYHA functional class III and an LVEF > 25% (n = 200) showed that all four variables, including anaerobic threshold, showed a statistically significant improvement in the CCM group as compared with in the controls. This suggested that CCM was perhaps more effective in patients with milder LV dysfunction and ultimately led to the completion of the FIX-HF-5C trial, which was confined to patients with an LVEF of 25% to 45%. Importantly, there was no difference in mortality between the CCM and control groups.

One hundred-sixty patients with NYHA functional class III/IV and an LVEF of 25% to 45% were randomized to continued maximal medical therapy or maximum medical therapy plus CCM and followed for 24 weeks. Peak VO_2_, NYHA functional class, six-minute walk test, and Minnesota Living with Heart Failure Questionnaire score results were assessed at 12 weeks and 24 weeks. There was a significant improvement in all four measures in the CCM group as compared with in those using medical treatment alone. The improvement in peak VO_2_ was 0.836 mL/kg/min for the CCM group, which is similar to the improvement in peak VO_2_ seen with cardiac resynchronization therapy (CRT) in the Safety and Effectiveness of CRT with Defibrillation (CONTAK CD) study (0.8 mL/kg/min).^4^

The FIX-HF-5C trial suggests a role for CCM in a specific subgroup of heart failure patients: symptomatic class III or ambulatory class IV patients on maximal medical therapy with an LVEF of 25% to 45% who are not candidates for CRT by virtue of having a relatively narrow QRS of < 130 ms. CCM appeared to increase myocardial performance without increasing mortality, unlike with the use of inotropic drugs. However, this therapy does not supplant an ICD—it supplements it.

## ENHANCE CRT

Another thought-provoking clinical trial presented was the CRT Implant Strategy Using the Longest Electrical Delay for Non-left Bundle Branch Block (LBBB) Patients (ENHANCE CRT) pilot study^5^ (NCT01983293). Again, the focus here was on nontraditional CRT candidates—in this case, patients with right bundle branch block (RBBB) or intraventricular conduction delay (IVCD). Several previous randomized trials, such as the Multicenter Insynch Randomized Clinical Evaluation (MIRACLE) trial, seemed to indicate that such patients had a lower response rate and reduced degree of response to CRT.^6,7^ ENHANCE CRT was aimed at seeing if targeted LV lead placement, determined by the longest QLV interval, would improve the response rate in non-LBBB patients. Recall that the TARGET trial^8^ has already shown that lead placement based on mechanically delayed LV segments is superior in LBBB patients.

Two hundred-forty-eight patients who met the criteria for implant according to the 2013 American Heart Association/Heart Rhythm Society guidelines were enrolled in this study. All had either RBBB or IVCD. These patients were randomized in a 2:1 distribution to targeted LV lead (placed at the latest activated LV site) or standard of care (SOC). All patients received a quadripolar system from St. Jude Medical (St. Paul, MN, USA), and none received multipoint pacing (MPP). The composite endpoint included death, heart failure hospitalization, NYHA functional class, and Patient Global Assessment score (with the latter consisting of a single question: are you improved, markedly improved, unchanged, worsened, or markedly worsened?).

One hundred-ninety of the 248 initially enrolled patients were available at 12 months for data analysis. Death and heart failure hospitalizations occurred at the same rate in the targeted lead placement and SOC groups. The response rates with respect to the above criteria were also similar (63.7% and 71.4%; p = 0.38). LVEF increased similarly between the two groups (5.5% ± 11% in the SOC arm and 5.8% ± 9.6% in the targeted lead placement arm; p = not significant).

Although ostensibly representing a negative result, this study’s findings raise a couple of interesting points. First, the response rate in the non-LBBB patients was between 64% and 72%, which compares very favorably with rates in older CRT studies in LBBB patients. This suggests that non-LBBB patients can benefit significantly from CRT.

The improved response rate in both arms may be due to the use of the quadripolar LV lead used in this study, which is different from the case of older CRT studies that relied on unipolar or bipolar leads. The availability of multiple electrodes may allow for pacing to occur from a more basal site versus what is allowed using conventional unipolar or bipolar leads. Indeed, in recent trials of LBBB patients, the response rate was 77% at three months in the MPP trial and 74% at six months in the Adaptive CRT trial.^9,10^ Both trials used quadripolar leads.

**Figure 2:**
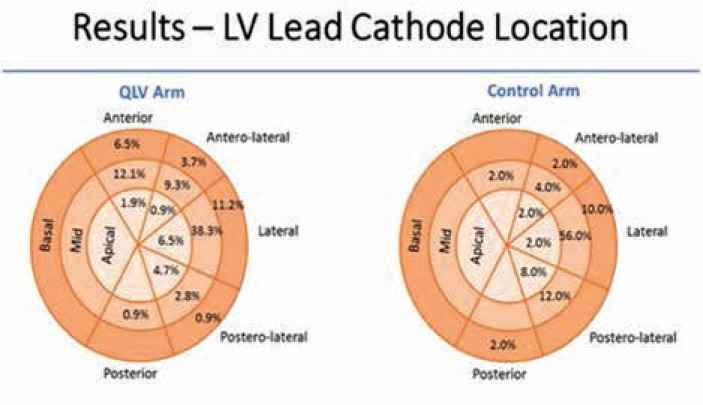
Distribution of LV pacing electrodes among the QLV and control arms in the ENHANCE CRT trial. Image reproduced with permission from Dr. Jagmeet P. Sing.

Of interest is also the fact that, in both the SOC and targeted lead placement arms, the large majority of leads were placed over the lateral wall **(Figure 2)**. This explains the lack of improvement seen with targeted LV lead placement. It seems that the lateral wall is the target in most patients with RBBB and IVCD as well!
